# Role of Lactate Dehydrogenase as a Biomarker of Early Cardiac Remodeling: A Cross-Sectional Study

**DOI:** 10.7759/cureus.68906

**Published:** 2024-09-07

**Authors:** Lejla Brigic, Ehlimana Mušija, Faris Kadić, Mirela Halilčević, Azra Durak-Nalbantic, Lejla Dervišević, Una Glamoclija

**Affiliations:** 1 Cardiology, Sarajevo University Clinical Center, Sarajevo, BIH; 2 Clinic for Heart, Blood Vessel and Rheumatoid Diseases, Sarajevo University Clinical Center, Sarajevo, BIH; 3 Cardiology, Dr. Abdulah Nakas General Hospital, Sarajevo, BIH; 4 Human Anatomy, Sarajevo University Clinical Center, Sarajevo, BIH; 5 Faculty of Pharmacy, University of Sarajevo, Sarajevo, BIH

**Keywords:** cardiac prevention, cardiac remodelin, ejection fraction, lactate dehydrogenase, myocardial infarction

## Abstract

Background: Lactate dehydrogenase (LDH) isoenzyme assay was used widely in the past to diagnose myocardial infarction (MI). Recent studies show that lactate dehydrogenase seems to be a promising biomarker of adverse left ventricular remodeling.

Objectives: Higher levels of these biomarkers were associated with lower odds for favorable reverse remodeling in patients with MI.

Methods: The study was performed on patients with the first occurrence of acute myocardial infarction (ST-elevation myocardial infarction (STEMI) or non-ST-elevation myocardial infarction (NSTEMI)), aged 34 to 80 years who underwent catheterization at the admission or during their hospital stay depending on indications. In this study, we compared peak levels of lactate dehydrogenase (LDH) and left ventricular ejection fraction (LVEF). Peak values of LDH were used from the second to the fourth day of hospitalization. Echocardiography has been done in the first 72 hours, which represents an early phase of cardiac remodeling. The ejection fraction was evaluated using the Simpson method.

Results: Spearman's rank test showed a negative, statistically significant correlation between LDH and ejection fraction ρ(80)=−0.543, p<0.001. Weighted least squares regression model included LDH concentration, age, and type of myocardial infarction (STEMI/NSTEMI), and the slope coefficient for the LDH level was −0.010 (95% confidence interval (CI): −0.013 to −0.006). With each unit of LDH increase, there was a decrease of 0.01% in left ventricular ejection fraction when age and type of myocardial infarction were held constant.

Conclusion: The increased LDH level could be a new predictor for early myocardial remodeling after the first occurrence of myocardial infarction independent of age and type of myocardial infarction.

## Introduction

The acute loss of myocardium results in an abrupt increase in loading conditions that induces a unique pattern of remodeling involving the infarcted border zone and remote non-infarcted myocardium. Myocyte necrosis and the resultant increase in load trigger a cascade of biochemical intracellular signaling processes that initiate and subsequently modulate reparative changes, which include dilatation, hypertrophy, and the formation of a discrete collagen scar [1].

Novel studies showed that measuring different biomarker concentrations early after acute myocardial infarction (AMI) might provide a cost-efficient and widely available tool to assess infarct severity, myocardial dysfunction, and clinical outcomes. Early identification of patients at high risk using biomarkers might, therefore, help to guide more individualized treatment strategies [2].

The use of biomarkers is very convenient for patients, as it is a widely available non-invasive, operator-independent method. Recent studies have shown that an old biomarker of cardiac necrosis, lactate dehydrogenase (LDH), can be used as a biomarker in the prognosis of cardiac remodeling [3]. Lactate dehydrogenase (LDH) is an important energy-metabolizing enzyme widely distributed in numerous tissues. It is released into the peripheral blood after cell damage and is often used to indicate cell damage. However, measuring LDH as a routine biochemical test in practice is often overlooked. Very little is known in the literature about the influence of LDH values on long-term changes in heart function after acute myocardial infarction [4].

The level of the LDH isoenzyme increases 24 to 72 hours after the occurrence of a myocardial infarction (MI) and reaches its maximum concentration after three to four days. The elevated level in the blood remains for eight to 14 days, which makes it a late biomarker of myocardial infarction [5]. LDH seems to be a promising biomarker of adverse, according to a study conducted by Wegiel et al. in 2021. On the other hand, higher levels of these biomarkers were associated with a reduced chance of occurrence of favorable reverse remodeling in MI patients [3].

This study aimed to prove a correlation between lactate dehydrogenase levels as a prognostic biomarker and ejection fraction (EF) in the early remodeling phase of acute myocardial infarction patients.

## Materials and methods

In this retrospective observational cross-sectional study total of 181 patients were screened who were diagnosed with acute myocardial infarction and admitted to the Intensive Care department of the Clinic for Heart, Vessel, and Rheumatoid Diseases in the University Clinical Center of Sarajevo. The study included patients with first occurrence ST-elevation myocardial infarction (STEMI) and non-ST-elevation myocardial infarction (NSTEMI), both genders aged 34 to 80, who underwent catheterization at admission or during their hospital stay, depending on indications. All the patients had measured lactate dehydrogenase levels during admission and several times during the hospital stay and echocardiography.

In our study, we excluded patients with incomplete data and conditions that additionally influence EF or levels of LDH, such as unstable angina, aortic stenosis, reinfarction or new onset of MI, and previous decreases in EF. Patients with acute coronary syndrome presented as unstable angina have been excluded since levels of LDH in such patients are constant when compared to STEMI/NSTEMI patients. Aortic stenosis can eventually cause myocardial hypertrophy, due to increased afterload, which can prograde into dilatation and cause a reduction in EF. Reinfarction or new onset of MI with already decreased EF will have an additional decrease in EF. Registered reduction or increase of EF due to other conditions, such as myocarditis and cardiomyopathies, before the onset of AMI, will additionally influence EF. After eliminating all patients with exclusion criteria, the study was conducted over 82 patients.

Peak values of lactate dehydrogenase were used from the second to the fourth day of hospitalization. Echocardiography is done in the first 72 hours, representing an early phase of cardiac remodeling. The ejection fraction has been estimated using the Simpson method.

The study was approved by the local ethics committee of the Clinical Center University of Sarajevo (ref number 51-30-5-46292/23) and performed in conformity with the ethical guidelines of the Declaration of Helsinki. Hospital admission and all data were collected between August 2022 and January 2023. Written informed consent was obtained from all patients before study inclusion.

Descriptive statistics (absolute numbers and percentages) were used for the presentation of patients' baseline data. The normality of data distribution was assessed visually by analyzing histograms, by calculation of the z-score for skewness and kurtosis, and by the Kolmogorov-Smirnov test. After visual inspection of the scatter plot evaluating the linear relationship between ejection fraction and LDH concentration, Spearman’s Rho test was conducted. Weighted least squares regression was run to evaluate how lactate dehydrogenase levels can be used to predict ejection fraction. The linearity of correlation was assessed by visualization of a scatter plot of ejection fraction versus lactate dehydrogenase levels using the ggplot2 package in R Statistical Software (Foundation for Statistical Computing, Vienna, Austria). Due to the heteroscedasticity of residuals, as visualized by the plot using standardized residuals versus standardized predicted value, square root and logarithmic transformation of ejection fraction were performed but did not give adequate results, so weighted least squares regression was used. Lactate dehydrogenase levels, age, and type of myocardial infarction (STEMI/NSTEMI) were included in the regression model. Statistical analysis was performed using the Statistical Package for Social Sciences (SPSS) program version 29.0 (IBM Corp., Armonk, New York, USA) [6].

## Results

A total of 82 patients were included in the study. Most patients have been male (79.3%), and most of them had STEMI. All the participants were 30 to 80 years old (Table 1).

**Table 1:** Baseline characteristics of patients included in the study. Data are presented as *mean ± standard deviation, or †absolute numbers (percentage of total number of patients). STEMI: ST-elevation myocardial infarction, NSTEMI: non-ST-elevation myocardial infarction.

Parameter	Patients (n=82)
Age, years*	62 ± 10
Gender, male^†^	65 (79%)
Type of myocardial infarction^†^	
STEMI	67 (82%)
NSTEMI	15 (18%)

The ejection fraction median value was 45.0% (IQR: 37.25-48.00%). The median LDH concentration was 4025 units/L (IQR: 264.3-713.5 units/L). 

Spearman's rank test showed that there is a negative, statistically significant correlation between LDH and ejection fraction in patients with acute coronary syndrome admitted to the intensive care department, ρ(80)=−0.543, p<0.001. 

A logarithmic transformation of the ejection fraction was performed. After the transformation of the dependent variable, the linearity of the correlation was checked by inspection of the scatter plot.

**Figure 1:**
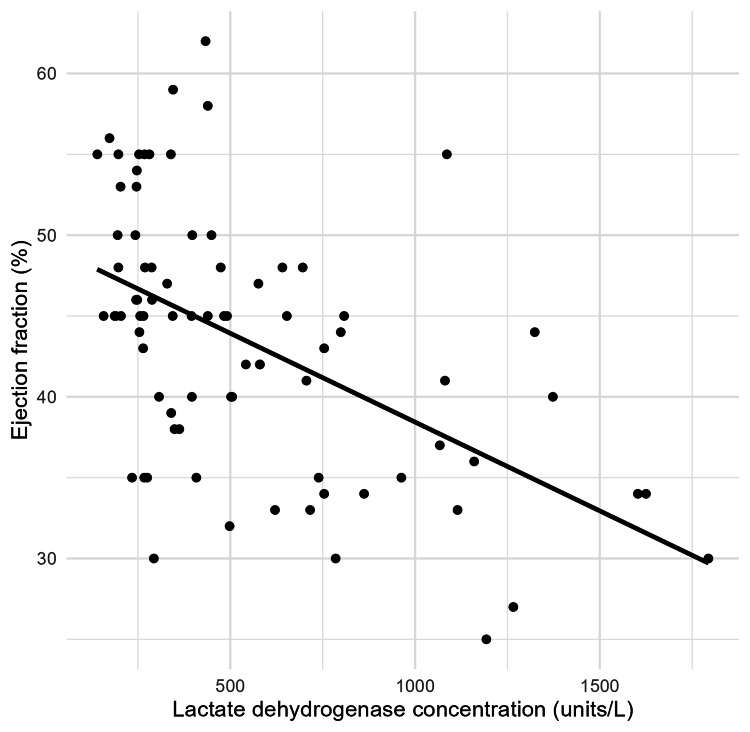
Scatter plot showing the linear relationship between lactate dehydrogenase and ejection fraction.

Weighted least squares regression model included LDH concentration, age, and type of myocardial infarction (STEMI/NSTEMI) and was statistically significant, F(3, 78)=13.55, p<0.001. The model accounted for 34.3% of the variation in ejection fraction with adjusted R^2^=31.7%, a substantial effect size according to Cohen's 1988 guidelines [6]. The LDH level was significantly negatively correlated with ejection fraction when age and type of myocardial infarction were held constant. The slope coefficient for the LDH level was −0.010 (95% confidence interval (CI) −0.013 to −0.006), so with each unit of LDH increase, there was a decrease of 0.01% of ejection fraction. This scatter plot shows that there is a moderate negative association between lactate dehydrogenase levels and ejection fractions (Figure 1). 

## Discussion

Acute myocardial infarction is one of the most serious cardiovascular diseases in the world. Acute loss of myocardium leads to a sudden increase in loading conditions that induces a unique pattern of remodeling involving the infarcted border zone and remote non-infarcted myocardium. Remodeling may be physiological and adaptive during normal growth or pathological due to myocardial infarction (as mentioned above), cardiomyopathy, hypertension, or valvular heart disease [1,7].

Unfavorable left ventricular remodeling after myocardial infarction is the leading cause of heart failure and one of the main causes of death. Remodeling is a set of different changes that occur not only at the molecular level in the cells but also in the interstitial tissue. All these events lead to changes in the mass, shape, size, and function of the left ventricle. An enlarged left ventricle and a decrease in the ejection fraction are indicators of a poor prognosis for the patient [8]. 

Measuring new and different biomarkers during the subacute or acute phase of myocardial infarction represents a simple and cost-effective model for assessing the risk of adverse left ventricular remodeling in the clinical setting. To date, over fifty biomarkers have been described that can be used to determine adverse left ventricular remodeling [9]. The LDH enzyme is present in numerous organs, and its elevated values can be found in numerous diseases. This fact may indicate that LDH values can serve as a reliable marker for a poorer prognosis in numerous diseases. Numerous studies have confirmed the association between high LDH values and prognosis in tumors, while a small number of studies have investigated the association of LDH values with cardiovascular diseases [10]. After the occurrence of a myocardial infarction, LDH begins to rise for nine to twenty hours and reaches its highest concentration after thirty-six to sixty days, but returns to reference values after six to ten days. In the heart, LDH values are a reliable enzymatic indicator of myocardial damage. Liao et al. found that elevated LDH values are associated with an increased rate of cardiovascular mortality in arsenic poisoning [11]. Wu et al. found a positive correlation between elevated LDH values and mortality rates in patients with metabolic syndrome [12]. Also, in a recent study by Matsumoto et al., it was found that high LDH concentrations were associated with a high rate of in-hospital mortality in cardiovascular patients suffering from the Coronavirus [13]. However, despite numerous studies, no study has yet been conducted that would determine the association of LDH values with structural changes in the myocardium in patients with cardiovascular diseases and the influence of LDH values on the prognosis of these changes.

This study was designed to analyze whether changes in LDH levels could help us predict adverse remodeling and poorer ejection fraction in patients with myocardial infarction. Considering the above, in our study, we determined the existence of a statistically significant negative correlation between the LDH value and the drop in the ejection fraction in patients after acute myocardial infarction. We found that the drop in LDH values correlates with an increased ejection fraction. According to recent research from Germany, Naumann et al. found that lactate dehydrogenase (LDH) isoform expression occurs in residual tissue after myocardial infarction in rats [14]. In everyday practice, troponin I has proven to be the most valuable marker that objectively indicates the degree of heart muscle necrosis. However, survival analyses showed LDH to be superior compared to troponin I or creatine kinase isoenzyme in assessing the effect on long-term myocardial structure and function in patients with acute myocardial infarction. LDH is an important enzyme in cellular metabolism because it catalyzes the conversion of purivate and lactate [15]. Compared to other organs, the heart muscle requires more energy to perform its constant function as a pump. However, after a heart attack, there is a strong restriction of numerous metabolic processes, a reduced amount of myocardial energy, and an increase in glycolysis and lactate levels. Altered myocardial metabolism can initiate undesirable cardiac muscle remodeling [16]. In the study by Zhang et al., it was found that longitudinal changes in cardiac function were independently associated with high LDH values in elderly patients with acute myocardial infarction. LDH values are superior in comparison with other myocardial biomarkers and can be used as a useful parameter in the assessment of myocardial dysfunction after a heart attack. To confirm the influence of LDH values on the cardiac structure and function of patients with acute myocardial infarction, Zhang et al. found that in elderly patients with or without revascularization therapy, the internal diameter of the left ventricle and volumes were larger in patients with elevated LDH values in comparison with patients who had normal LDH values. These results agree with our study, again suggesting that elevated LDH values can be used as a predictor of adverse left ventricular remodeling in patients after myocardial infarction [17]. Dai et al. confirmed the role of elevated LDH values in the development of cardiac hypertrophy under hemodynamic stress in an animal model [18]. It is believed that the value of LDH can also be an important predictor of other heart diseases. Studies have proven that LDH values are an important prognostic factor in children with myocarditis [19]. Our results showed that increased myocardial LDH activity was correlated with impaired left ventricular diastolic function measured as a decrease in ejection function. These results are from other studies, where LDH levels were high in patients with end-stage heart failure but also patients with valvular heart disease, parallel to decreasing systolic and diastolic ventricular function. Piper et al. found that LDH values were increased not only in patients with terminal heart failure but also in patients with valvular diseases simultaneously with a decline in systolic and diastolic function of the ventricles. A significant change in LDH isoenzymes was also found in patients with terminal heart failure, but not in patients with valvular damage. It is also found that LDH values will increase faster than the values of LDH isoenzymes in patients with left ventricular dysfunction [20]. LDH activity but not the shift in LDH isozyme values can be used to detect early energy imbalance and as a marker for myocardial maladaptation under chronic stress [21]. Baykiz et al. found that in recovering COVID-19 patients, high LDH levels were associated with reduced left ventricular global longitudinal strain during follow-up visits. This indicates that patients could have benefited from early identification of subclinical heart muscle injury by measuring LDH levels after hospitalization [22]. Serum LDH values are a useful predictive indicator in hemodialysis patients. This marker enables clinicians to intervene and prevent possible arrhythmias and sudden cardiac death by early detection of changes in its values [23]. Zu et al. stated that a high level of LDH is associated with arterial stiffness and cardiovascular disease, and it could be used as a new marker in health-examined populations [24]. In the study of Węgiel et al., LDH and MR-proADM appeared to be reliable indicators of unwanted remodeling, where high concentrations of these biomarkers were associated with a reduced chance of desired remodeling in patients after myocardial infarction [3]. A prospective follow-up study from Hamada et al. showed that constantly elevated LDH values in patients with hypertrophic cardiomyopathy are associated with myocardial damage that eventually leads to death due to heart failure. Also, the progression of unfavorable left ventricular remodeling was faster and more severe in patients who had higher LDH values [25].

Lactate dehydrogenase consists of two subcomponents: heart (H) and muscle (M). Its activity in heart muscle is hundreds of times higher than in normal serum. Because of this, when the heart muscle is damaged, LDH concentrations increase significantly. In our study, we proved that patients who have a lower ejection fraction, that is, greater damage to the heart muscle, have significantly elevated LDH values in the serum. This confirms the hypothesis that LDH can be used in daily clinical practice as a useful and reliable biomarker for risk assessment in patients with cardiovascular disease. Compared with the results of the studies previously mentioned, our study reveals the effects that the serum LDH value has on the risk assessment for the occurrence of unfavorable myocardial remodeling after myocardial infarction. LDH, which is normally present in various tissues and cells, is released into the blood, resulting in elevated serum levels. If there is an increase in LDH to a certain extent, this may indicate serious tissue damage not only to the heart but also to other organs, which is consistent with our results that show elevated LDH values associated with weaker myocardial function. Biomarkers such as LDH may allow us an early prediction of left ventricular remodeling that could easily be used in a broad range of patients with myocardial infarction.

Although the results of our study show reliable results, there are still certain limitations that we observed when conducting our study. The main limitation of this study is that, besides initially having a large sample, exclusion criteria were high, so we ended with a small sample size. Due to the mentioned, we also observed patients with large age differences and included both STEMI and NSTEMI patients, which should, in further larger studies, be divided into two samples due to possible bias. The collection of LDH samples was during the period (two days) not in a single moment because oscillation in LDH is not exact in every individual and this study included only peak values. There are studies proposing multi-biomarkers, but also studies that are presenting various arguments against multi-testing and prefer single-marker approach [2,26,27]. We used the second one. For most of the patients echo data, showing the ejection fraction of patients before admission, was missing. Also, we used echocardiography for the assessment of EF due to guidelines and cost efficiency, although we are familiar with the golden standard for EF estimation, which is magnetic resonance. Changes in EF as the main representative of cardiac remodeling were examined in this study but for further research assessment of end-systolic and end-diastolic volumes of LV, heart size, shape, and mass should be done as well.

## Conclusions

Our data indicate that the level of LDH activity is increased when ejection fraction is lower in patients with acute coronary syndrome admitted to the intensive care department. A significant shift of the LDH levels could be used for early phase cardiac remodeling prognosis in acute myocardial infarction independent of age and type of myocardial infarction. However, further studies on larger groups of patients are necessary to provide more information in this regard. Biomarkers, such as LDH, offer a non-invasive way to predict the condition of patients with myocardial infarction. In this way, economic savings are achieved, and greater patient comfort is obtained. The persistent elevation of cardiac enzymes such as LDH indicated ongoing myocardial injury that could ultimately result in death by heart failure. We conclude that the progression of remodeling is more rapid and severe in patients with higher plasma LDH levels.
